# Impact of 30-day prescribed opioid dose trajectory on fatal overdose risk: A population-based, statewide cohort study

**DOI:** 10.1007/s11606-023-08419-6

**Published:** 2023-10-04

**Authors:** Stephen G. Henry, Shao-You Fang, Andrew J. Crawford, Garen J. Wintemute, Iraklis Erik Tseregounis, James J. Gasper, Aaron Shev, Abigail R. Cartus, Brandon D.L. Marshall, Daniel J. Tancredi, Magdalena Cerdá, Susan L. Stewart

**Affiliations:** 1 University of California Davis Center for Healthcare Policy and Research; University of California, Davis, California, Sacramento USA; 2 Department of Internal Medicine, University of California, Davis, California, Sacramento USA; 3 Violence Prevention Research Program; University of California, Davis, California, Sacramento USA; 4 Department of Emergency Medicine, University of California, Davis, California, Sacramento USA; 5 Department of Family and Community Medicine, University of California, San Francisco, California, San Francisco USA; 6 Department of Epidemiology, Brown University School of Public Health, Rhode Island, Providence USA; 7 Department of Pediatrics, University of California, Davis, California, Sacramento USA; 8 Department of Population Health, Center for Opioid Epidemiology and Policy; New York University Grossman School of Medicine, New York City, New York USA; 9 Department of Public Health Sciences, University of California, Davis, California, Davis USA

**Keywords:** opioid analgesics, drug overdose, opiate overdose, drug tapering, prescription drug monitoring programs, controlled substances, risk factors

## Abstract

**Background:**

Both increases and decreases in patients’ prescribed daily opioid dose have been linked to increased overdose risk, but associations between 30-day dose trajectories and subsequent overdose risk have not been systematically examined.

**Objective:**

To examine the associations between 30-day prescribed opioid dose trajectories and fatal opioid overdose risk during the subsequent 15 days.

**Design:**

Statewide cohort study using linked prescription drug monitoring program and death certificate data. We constructed a multivariable Cox proportional hazards model that accounted for time-varying prescription-, prescriber-, and pharmacy-level factors.

**Participants:**

All patients prescribed an opioid analgesic in California from March to December, 2013 (5,326,392 patients).

**Main Measures:**

Dependent variable: fatal drug overdose involving opioids. Primary independent variable: a 16-level variable denoting all possible opioid dose trajectories using the following categories for current and 30-day previously prescribed daily dose: 0-29, 30-59, 60-89, or ≥90 milligram morphine equivalents (MME).

**Key Results:**

Relative to patients prescribed a stable daily dose of 0-29 MME, large (≥2 categories) dose increases and having a previous or current dose ≥60 MME per day were associated with significantly greater 15-day overdose risk. Patients whose dose decreased from ≥90 to 0-29 MME per day had significantly greater overdose risk compared to both patients prescribed a stable daily dose of ≥90 MME (aHR 3.56, 95%CI 2.24-5.67) and to patients prescribed a stable daily dose of 0-29 MME (aHR 7.87, 95%CI 5.49-11.28). Patients prescribed benzodiazepines also had significantly greater overdose risk; being prescribed Z-drugs, carisoprodol, or psychostimulants was not associated with overdose risk.

**Conclusions:**

Large (≥2 categories) 30-day dose increases and decreases were both associated with increased risk of fatal opioid overdose, particularly for patients taking ≥90 MME whose opioids were abruptly stopped. Results align with 2022 CDC guidelines that urge caution when reducing opioid doses for patients taking long-term opioid for chronic pain.

**Supplementary Information:**

The online version contains supplementary material available at 10.1007/s11606-023-08419-6.

## INTRODUCTION

Prescribed opioid dose has long been recognized as an important risk factor for opioid-related overdose deaths. The initial studies that raised safety concerns about opioid analgesics all identified higher prescribed daily dose (measured in milligram morphine equivalents, MME) as a significant overdose risk factor.^1–3^ In response to these and other studies, the Centers for Disease Control and Prevention (CDC) issued guidelines in 2016 that discouraged clinicians from prescribing opioids for pain and strongly discouraged prescribing high doses.^4^ These guidelines catalyzed shifts in clinical practice away from opioid prescribing and led health systems, health insurers, and state lawmakers to impose dose-based restrictions on opioid prescribing.^5–8^

Shifts away from overprescribing were needed to reduce rates of opioid use disorder and overdose. However, recent studies indicate that decreasing patients’ prescribed opioid dose is also risky and is associated with increased rates of overdose,^9–11^ suicide,^12, 13^ and disruptions in care,^14, 15^ particularly for patients with physical opioid dependence or whose prescriptions are stopped abruptly. Unfortunately, most studies have limited power to examine how changes in prescribed dose affect overdose risk because fatal overdose events are rare among all patients prescribed opioids. Given this limitation, some studies have grouped patients based on long-term trajectories in their prescribed daily opioid dose.^16–18^ Most prior multivariable studies of overdose risk have analyzed opioid dose using mean daily dose as a either a categorical^19–22^ or binary^23^ variable; others have used total dose,^24–26^ maximum daily dose,^27^ or dose variability^28^ over a specific time period.

To our knowledge, no prior studies have examined the impact of patients’ short-term opioid dose trajectories on overdose risk across the full range of clinically important dose categories. As noted above, prior studies indicate that receipt of high-dose opioids as well as both increases *and* decreases in prescribed opioid dose can impact overdose risk in multiple ways that are difficult to disentangle without huge sample sizes. For example, dose increases can worsen opioid-related respiratory depression, while decreases can precipitate withdrawal or prompt risky behavior among patients with opioid use disorder. We conducted a large statewide cohort study to provide better data to clinicians and policymakers on how short-term changes in opioid dose affect overdose risk to help them make safer clinical decisions and design more nuanced prescribing policies, particularly for patients prescribed opioids for chronic pain.

## METHODS

### Overview

We analyzed statewide time-to-event data in a cohort comprising all patients ages 12 and older with any opioid prescription recorded in California’s prescription drug monitoring program database between March 1 and December 31, 2013. Our primary independent variable was a categorical, time-varying indicator denoting the trajectory of patients’ prescribed daily opioid dose over the previous 30 days. Our dependent variable was fatal opioid overdose during the subsequent 15 days measured using California death certificate records. Patients’ trajectories and outcomes were assessed in 15-day intervals through December 31, 2013. We chose 2013 because, prior to the 2016 CDC guidelines, both overall opioid prescribing and variation in prescribed opioid dose were much greater than they are today, increasing our ability to examine the inherent risks associated with dose changes. Moreover, most opioid-related deaths in 2013 involved prescription rather than illicit opioids; overdoses involving illicit synthetic opioids, particularly fentanyl, were rare in California before 2016.^29^

This study was approved by the California Committee for the Protection of Human Subjects and the University of California, Davis Institutional Review Board.

### Data sources

Controlled substance prescription data were obtained from California's prescription drug monitoring program, which contains records for all outpatient Schedule II-IV prescriptions dispensed in California. In addition to prescriber and pharmacy identifiers, each prescription record included date dispensed, National Drug Codes, quantity, strength per unit, days’ supply, patient sex, date of birth, name, and address.

Statewide death certificate records were obtained from the California Department of Public Health. Each record was linked to the CDC’s Multiple Cause of Death file, which assigns one ICD-10 code for the underlying cause of death and up to 20 additional ICD-10 codes for contributing causes of death to each death certificate.

### Data linkage

We linked 2013 death certificate and prescription drug records using The Link King,^30^ a publicly available SAS record linkage program that performs deterministic and probabilistic linkage^31^ and performs well on prescription drug monitoring program data.^32^ We first assigned a unique identifier to each death record and then identified all prescription records that matched to each death record. We then linked all prescription records not already associated with a death record that the program identified as belonging to the same person. We used the resulting patient-level file to identify our study cohort.

### Cohort construction

**Figure 1:**
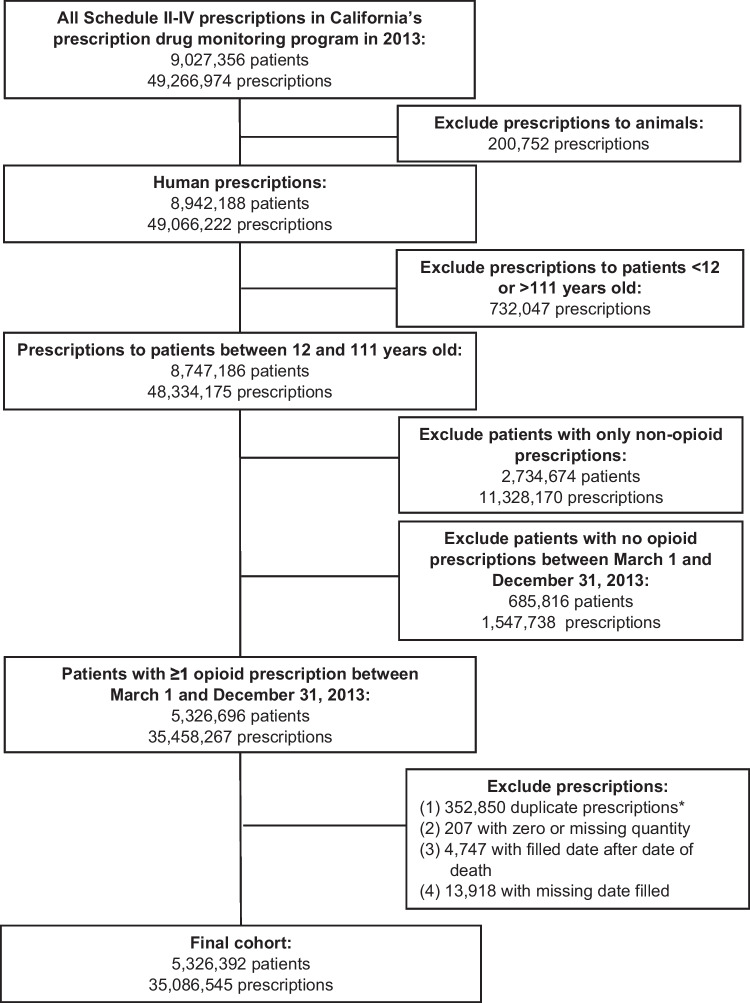
Identification of all patients in California who received one or more opioid prescription between March 1 and December 31, 2013. *Duplicate prescriptions were defined as prescriptions for the same person that had identical National Drug Codes, quantity, days’ supply, and fill date.

Our cohort included all patients in California who filled any Schedule II-IV opioid analgesic prescription between March 1 and December 31, 2013, and who were between 12 and 111 years old when they filled their first prescription. Data from other projects^33^ indicate that 98% of opioid prescriptions in November and December 2012 had days’ supply ≤30. We therefore chose March 1, 2013, as the earliest inclusion date because it was the first date for which we had complete data on prescribed opioid dose for both the date of assessment and the date 30 days previously. We excluded prescriptions to animals, duplicate prescriptions, prescriptions with missing fill date, with missing or zero quantity, or prescriptions filled after a patient’s date of death (see Fig. 1).

### Variable construction

Our dependent variable was a drug overdose death involving any opioids as defined by the CDC^34^: specifically, any underlying cause of death code indicating drug poisoning (X40-X44, X60-X64, X85, Y10-Y14) and any multiple cause-of-death code indicating opioids (T40.0, T40.1, T40.2, T40.3, T40.4, or T40.6). In a sensitivity analysis, we used overdoses involving prescription opioids (i.e., by excluding overdoses that involved heroin, T40.1) as the dependent variable. Patient status and all time-varying independent variables were assessed in 15-day increments.

Our primary, time-varying independent variable was prescribed daily opioid dose. We calculated patients’ daily dose using standard conversion factors^4^ to calculate total prescribed dose in MME and then dividing that total by the prescription’s days’ supply. When patients had multiple active opioid prescriptions, we summed the daily dose for all prescriptions. Consistent with CDC recommendations, we set the conversion factor for buprenorphine (2% of all opioid prescriptions) to zero because of its unique pharmacodynamics and risk profile.^4, 35^ Finally, we grouped daily dose into 4 ordinal categories: 0-29 MME, 30-59 MME, 60-89 MME, and ≥90 MME. To evaluate the impact of 30-day prescribed opioid dose trajectories, we created a 16-level variable for all possible combinations of patients’ current daily dose and their daily dose 30 days previously, using the same 4 categories for each.

We identified additional independent variables through a literature review and our own prior work.^33, 36^ Patient sex (male, female, unknown) and age (12-24, 25-64, ≥65) were analyzed as fixed variables. All other variables were time varying.

Additional opioid-related independent variables included indicators for whether patients had 1) any active opioid prescription, 2) two or more active opioid prescriptions, 3) any active prescription for a long-acting opioid formulation, 4) any active prescription for a liquid opioid formulation, and 5) any active prescription for a transdermal opioid (i.e., non-pill, non-liquid) formulation. A categorical variable for opioid type (hydrocodone, oxycodone, morphine, buprenorphine, codeine, fentanyl, methadone, hydromorphone, other, multiple types) was also included. Tramadol was not added to Schedule IV until 2014 and so was not included in our study data.

To account for risks associated with receiving opioids from multiple prescribers and pharmacies, we included indicator time-varying variables identifying whether patients had active prescriptions 1) written by two or more different prescribers and 2) filled by two or more different pharmacies.

To account for risks associated with concomitant use of other controlled substances, we included time-varying indicator variables identifying whether patients had an active prescription for 1) any benzodiazepine, 2) carisoprodol, 3) any psychostimulant, and 4) any Z drug (zolpidem, eszopiclone, or zaleplon).^37^ We included a 4-level variable describing the combination of patient’s current benzodiazepine status with their status 30 days previously.

### Statistical analysis and model construction

We began by examining monthly counts of our outcome variable versus the month of each patient’s first opioid prescription and the distribution of independent variables among patients who did versus did not experience a fatal opioid overdose.

We then constructed a time-varying Cox proportional hazards model to examine the relationships between 30-day prescribed opioid dose trajectory and fatal overdose risk. Follow up time was measured from the day of each patient’s first opioid dose on or after March 1, 2013, until the patient first experienced the outcome, died of some other cause, or reached the end of the study period. The follow up period ranged from 0 to 305 days.

To construct the model, we first added patient age, sex, and 30-day prescribed opioid dose trajectory as independent variables. We then added other opioid-related prescription-level variables, prescriber- and pharmacy-level variables, and finally, variables for other controlled substance prescriptions. We parameterized the model based on avoiding multicollinearity and optimizing overall model fit by minimizing Akaike information criterion. We explored models that measured daily opioid dose using both quantity of pills and MME per day; MME-based models were superior. We chose cutoffs for ordinal opioid dose categories that reflected clinically relevant dose intervals; models with categories for higher doses (e.g., ≥120 MME) had wide confidence intervals and poor model fit due to influence from outlier values. When adding a new variable caused major changes in other parameter estimates, we examined variable distributions to determine why the estimates changed. The optimal parameterization for many independent variables was binary indicator variables. For example, parameterizing overlapping prescriptions using number of days of overlap did not improve model fit compared to using a binary variable indicating whether patients had two or more active opioid prescriptions. We constructed separate tables of parameter estimates for the 16-level trajectory variable to facilitate examining the impact of 30-day change in daily opioid dose on overdose risk. We used the same model for both primary and sensitivity analyses.

Data preparation and analyses were conducted using SAS 9.4 and R 4.2.1.

## RESULTS

Figure 1 shows the flow chart for cohort construction. Our cohort comprised 5,326,392 patients who filled ≥1 opioid prescription between March 1 and December 31, 2013. Fewer than 1% of prescriptions had missing or duplicate data.

Over the study period, we recorded 797 overdose deaths involving opioids and 108,352 deaths from other causes. Table 1 shows the distribution of overdose deaths by month, stratified by the month of each patient’s first opioid dose. Nearly two-thirds (66%) of all overdoses occurred among patients who received their initial opioid prescription in March; 15% of all overdoses occurred among patients who received their first opioid prescription in April. As shown in Table 2, patients who died from an opioid-related overdose were more likely to be male and less likely to be age 65 years or older, compared to patients who did not. Approximately 22% of patients who died from an overdose were prescribed ≥90 MME per day both at the last assessment before their overdose and 30 days previously, compared to only 4% of patients who did not. Among patients who died from an overdose, 26% were not prescribed any opioids at either the last assessment before their overdose or 30 days previously Table 3.

**Table 1:** Distribution of month of first prescription, month of death, and cause of death among California patients with at least one opioid prescription between March 1 and December 31, 2013 (N=5,326,392 patients)

	Overdose death involving opioids (n=797)												Other causes of death (n=108,352)		Survived (n=5,217,243)	
	Month of death										Total					
Month of first pre-scription	Mar	Apr	May	Jun	Jul	Aug	Sep	Oct	Nov	Dec	n	%	n	%	n	%
Mar	39	44	57	67	57	55	55	48	58	44	524	65.75	42,320	39.06	1,308,422	25.08
Apr	0	8	17	16	9	19	11	16	11	13	120	15.06	16,677	15.39	687,690	13.18
May	0	0	7	4	4	10	6	4	6	3	44	5.52	11,252	10.38	518,853	9.94
Jun	0	0	0	2	4	1	4	3	3	6	23	2.89	8,577	7.92	438,764	8.41
Jul	0	0	0	0	7	5	3	5	3	8	31	3.89	7,489	6.91	439,774	8.43
Aug	0	0	0	0	0	3	5	5	4	6	23	2.89	6,494	5.99	413,721	7.93
Sep	0	0	0	0	0	0	4	4	0	5	13	1.63	5,248	4.84	363,603	6.97
Oct	0	0	0	0	0	0	0	3	2	3	8	1.00	4,700	4.34	371,764	7.13
Nov	0	0	0	0	0	0	0	0	4	4	8	1.00	3,583	3.31	333,182	6.39
Dec	0	0	0	0	0	0	0	0	0	3	3	0.38	2,012	1.86	341,470	6.55

**Table 2:** Patient and prescription characteristics among all analyzing samples, stratified by opioid overdose death among patients in California, March-December 2013, in the time-varying Cox proportional hazards model

Characteristics	All analyzing samples			
	Total		Opioid overdose death	No opioid overdose death
	n	%*	%^†^	%^‡^
Total number	68,829,519	100.00	797	68,828,722
Fixed covariates				
Patient age				
12-24	6,724,347	9.77	4.27	9.77
25-64	45,463,059	66.05	86.07	66.05
65 or more	16,642,113	24.18	9.66	24.18
Patient sex				
Female	39,828,044	57.86	43.41	57.86
Male	28,983,987	42.11	56.46	42.11
Unknown	17,488	0.03	0.13	0.03
Time-varying covariates				
Opioid dose				
Current daily dose (MME)				
0-29	55,996,030	81.35	45.42	81.36
30-59	6,415,511	9.32	12.92	9.32
60-89	2,541,436	3.69	9.79	3.69
≥90	3,876,542	5.63	31.87	5.63
Previous daily dose (30 days, MME)				
0-29	56,493,001	82.08	50.69	82.08
30-59	6,117,831	8.89	11.29	8.89
60-89	2,450,735	3.56	8.78	3.56
≥90	3,76,952	5.47	29.23	5.47
Current*Previous daily dose (MME)				
0-29*0-29	51,061,746	74.19	35.88	74.19
0-29*30-59	3,221,022	4.68	2.51	4.68
0-29*60-89	909,744	1.32	2.01	1.32
0-29*≥90	803,518	1.17	5.02	1.17
30-59*0-29	3,554,544	5.16	5.40	5.16
30-59*30-59	2,425,035	3.52	5.77	3.52
30-59*60-89	276,744	0.40	0.88	0.40
30-59*≥90	159,188	0.23	0.88	0.23
60-89*0-29	997,631	1.45	2.89	1.45
60-89*30-59	290,407	0.42	1.00	0.42
60-89*60-89	1,020,073	1.48	4.39	1.48
60-89*≥90	233,325	0.34	1.51	0.34
≥90*0-29	879,080	1.28	6.52	1.28
≥90*30-59	181,367	0.26	2.01	0.26
≥90*60-89	244,174	0.35	1.51	0.35
≥90*≥90	2,571,921	3.74	21.83	3.74
Other Opioid Characteristics				
Current active opioid prescription	19,974,572	29.02	64.24	29.02
More than one opioid prescription	3,110,890	4.52	21.71	4.52
Any long-acting opioid	3,431,932	4.99	27.85	4.99
Liquid formulation	244,929	0.36	1.00	0.36
Patch or other formulation	1,015,929	1.48	6.02	1.48
Opioid type				
Only hydrocodone or no use	61,830,883	89.83	59.72	89.83
Only oxycodone	2,014,844	2.93	9.16	2.93
Only codeine	1,169,914	1.70	0.63	1.70
Only morphine	570,444	0.83	4.77	0.83
Only Buprenorphine	365,589	0.53	1.38	0.53
Only Methadone	353,367	0.51	5.77	0.51
Only Fentanyl	260,019	0.38	1.38	0.38
Only Hydromorphone	173,944	0.25	1.51	0.25
Other opioid type	135,058	0.20	0.50	0.20
More than one opioid types	1,955,457	2.84	15.18	2.84
Pharmacy/Prescriber Characteristics				
≥2 pharmacies dispensing opioid prescriptions	808,071	1.17	4.64	1.17
≥2 prescribers prescribing opioid prescriptions	771,856	1.12	5.40	1.12
Other Controlled Substances				
Current active benzodiazepine prescription	7,287,761	10.59	42.41	10.59
Previous 30 days active benzodiazepine prescription	7,116,221	10.34	43.04	10.34
Current*Previous benzodiazepine prescription				
No*No	59,039,312	85.78	49.44	85.78
No*Yes	2,502,446	3.64	8.16	3.64
Yes*No	2,673,986	3.88	7.53	3.88
Yes*Yes	4,413,775	6.70	34.88	6.70
Current Z drug prescription	3,002,656	4.36	8.53	4.36
Current carisoprodol prescription	1,701,443	2.47	7.28	2.47
Current psychostimulant prescription	1,037,515	1.51	2.13	1.51

MME = milligram morphine equivalents

* Denominator is number of all analyzing samples (N=68,829,519)

† Denominator is number of opioid fatal overdose (n=797)

‡ Denominator is number of non-opioid fatal overdose (including other causes of death and survive, n=68,828,722)

**Table 3:** Estimated associations between monthly opioid dose and fatal opioid overdose (n = 797) among patients in California, March-December 2013 (N=68,829,519 analyzing samples)

Characteristics	All-opioid fatal overdose (n=792)		
	aHR*	95% CI	*P* value
Fixed covariates			
Patient age			
12-24	Ref	--	--
25-64	1.41	0.99-2.00	0.059
65 or more	0.42	0.28-0.64	<.001
Patient sex^†^			
Female	Ref	--	--
Male	1.86	1.61-2.14	<.001
Time-varying covariates			
Opioid Dose			
Current*Previous daily dose (MME)			
0-29*0-29	Ref	--	--
0-29*30-59	1.26	0.77-2.06	0.354
0-29*60-89	3.19	1.90-5.37	<.001
0-29*≥90	7.87	5.49-11.28	<.001
30-59*0-29	1.25	0.84-1.86	0.270
30-59*30-59	1.41	0.96-2.08	0.080
30-59*60-89	1.75	0.79-3.88	0.166
30-59*≥90	2.55	1.16-5.62	0.020
60-89*0-29	1.98	1.22-3.20	0.006
60-89*30-59	1.70	0.81-3.56	0.162
60-89*60-89	2.08	1.35-3.19	0.001
60-89*≥90	2.72	1.45-5.12	0.002
≥90*0-29	3.35	2.24-5.01	<.001
≥90*30-59	4.13	2.30-7.41	<.001
≥90*60-89	2.23	1.18-4.25	0.014
≥90*≥90	2.21	1.52-3.21	<.001
Other Opioid Characteristics			
Current active opioid prescription (Ref=no)	1.63	1.22-2.20	0.001
More than one opioid prescription (Ref=0-1)	1.15	0.82-1.63	0.421
Any long-acting opioid (Ref=no)	1.16	0.84-1.62	0.370
Liquid formulation (Ref=no)	0.77	0.37-1.62	0.493
Patch or other formulation (Ref=no)	1.03	0.69-1.53	0.889
Opioid type			
Only Hydrocodone or no use	Ref	--	--
Only Oxycodone	1.42	1.04-1.92	0.025
Only Codeine	0.47	0.19-1.14	0.094
Only Morphine	2.33	1.47-3.70	<0.001
Only Buprenorphine	1.55	0.73-3.28	0.255
Only Methadone	3.54	2.19-5.72	<.001
Only Fentanyl	1.71	0.80-3.64	0.167
Only Hydromorphone	2.59	1.43-4.69	0.002
Other opioid type	1.31	0.48-3.59	0.605
More than one opioid type	1.40	0.96-2.05	0.082
Pharmacy/Prescriber Characteristics			
Number of pharmacies dispensing opioid prescriptions (Ref=0-1)	0.76	0.52-1.11	0.152
Number of prescribers prescribing opioid prescriptions (Ref=0-1)	1.26	0.87-1.82	0.223
Other Controlled Substances			
Current*Previous active benzodiazepine prescription			
No*No	Ref	--	--
No*Yes	3.23	2.44-4.28	<.001
Yes*No	2.30	1.71-3.09	<.001
Yes*Yes	5.16	4.27-6.25	<.001
Current Z drug prescription (Ref=no)	0.99	0.77-1.29	0.966
Current carisoprodol prescription (Ref=no)	0.93	0.70-1.23	0.611
Current psychostimulant prescription (Ref=no)	0.65	0.40-1.06	0.085

MME = milligram morphine equivalents; aHR = adjusted hazards ratio; 95% CI = 95% confidence interval

* Time-varying Cox proportional hazards model; model adjusts for all listed covariates; parameters that are statistically significant at the *P* = 0.05 level are in bold.

† Category of unknown sex (n = 17,488, 0.03%) not shown

Tables 4a summarizes overdose risk associated with 30-day prescribed opioid dose trajectories relative to a stable, low dose of 0-29 MME per day. Thirty-day dose increases from 0-29 MME per day to either 60-89 MME (aHR 1.98, 95%CI 1.22, 3.20) or ≥90 MME (aHR 3.35, 95%CI 2.24, 5.01) per day as well as from 30-59 MME to ≥90 MME per day (aHR 4.13, 95%CI 2.30, 7.41) were all associated with significantly increased overdose risk during the subsequent 15 days, as was having either a previous or current dose ≥60 MME per day (with one exception; the increased risk associated with a 30-day dose decrease from 60-89 MME to 30-59 MME per day was not significant; aHR 1.75, 95%CI 0.79-3.88).

**Table 4:** Comparison of overdose risk based on current and previous prescribed daily opioid dose from the multivariable Cox proportional hazards model*

**4a**. Overdose risk relative to patients with current and prescribed dose of 0-29 MME									
		Previous daily dose (30 days, MME)							
		0-29		30-59		60-89		≥90	
		aHR	95% CI	aHR	95% CI	aHR	95% CI	aHR	95% CI
Current daily dose (MME)	0-29	1.0	--	1.26	(0.77, 2.06)	**3.19**	(**1.90, 5.37**)	**7.87**	(**5.49, 11.28**)
	30-59	1.25	(0.84, 1.86)	1.41	(0.96, 2.08)	1.75	(0.79, 3.88)	**2.55**	(**1.16, 5.62**)
	60-89	**1.98**	(**1.22, 3.20**)	1.70	(0.81, 3.56)	**2.08**	(**1.35, 3.19**)	**2.72**	(**1.45, 5.12**)
	≥90	**3.35**	(**2.24, 5.01**)	**4.13**	(**2.30, 7.41**)	**2.23**	(**1.18, 4.25**)	**2.21**	(**1.52, 3.21**)
**4b**. Overdose risk relative to patients with the same previous prescribed dose and no change in their current prescribed dose									
		Previous daily dose (30 days, MME)							
		0-29		30-59		60-89		≥90	
		aHR	95% CI	aHR	95% CI	aHR	95% CI	aHR	95% CI
Current daily dose (MME)	0-29	1.00	--	0.89	(0.49, 1.63)	1.54	(0.79, 2.98)	**3.56**	(**2.24, 5.67**)
	30-59	1.25	(0.84, 1.86)	1.00	--	0.84	(0.37, 1.91)	1.16	(0.53, 2.51
	60-89	**1.98**	(**1.22, 3.20**)	1.20	(0.57, 2.55)	1.00	--	1.23	(0.68, 2.24)
	≥90	**3.35**	(**2.24, 5.01**)	**2.92**	(**1.63, 5.31**)	1.08	(0.55, 2.09)	1.00	--

MME = milligram morphine equivalents; aHR = adjusted hazards ratio; 95% CI = 95% confidence interval

*Bold font indicates adjusted hazard ratios that are statistically different than the reference category at the *P* = 0.05 level.

Table 4b summarizes overdose risk associated with 30-day prescribed opioid dose trajectories relative to patients with the same previous dose and no change in dose over 30 days. Increases from 0-29 MME per day to either 60-89 MME or ≥90 MME per day were associated with significantly greater overdose risk (the reference category and parameter estimates for these patients were identical to those in Table 4a). The overdose risk associated with a 30-day increase from 30-59 MME to ≥90 MME per day also remained significant (aHR 2.92, 95%CI 1.63, 5.31). For patients with a previous dose of ≥60 MME per day, large dose reductions (≥2 categories) were all associated with increased overdose risk, but only the increased risk associated with a decrease from ≥90 MME to 0-29 MME per day was statistically significant compared to patients on a stable dose. Patients whose opioid dose decreased from ≥90 to 0-29 MME per day had a significantly greater overdose risk during the subsequent 15 days compared to both patients prescribed a stable daily dose of ≥90 MME (aHR 3.56, 95%CI 2.24-5.67) and to patients prescribed a stable daily dose of 0-29 MME (aHR 7.87, 95%CI 5.49-11.28). Thirty-nine of the 40 patients who had a fatal overdose associated with this trajectory had their opioid access interrupted completely (i.e., current dose = 0 MME per day) after previously being prescribed ≥90 MME per day.

Patients prescribed benzodiazepines had substantially greater overdose risk; patients prescribed benzodiazepines at their current assessment or 30 days previously had significantly greater risk than patients prescribed benzodiazepines at neither time point. Being prescribed Z-drugs, carisoprodol, or prescription stimulants was not associated with significantly greater overdose risk. Overall results for our sensitivity analysis excluding overdose deaths involving heroin were similar to the primary findings (Table S1).

## DISCUSSION

This project examined the impact of 30-day prescribed opioid dose trajectories on patients’ risk of fatal opioid-related overdose for all patients in California in 2013. For patients prescribed low daily opioid doses, large dose increases (≥2 categories) over 30 days were associated with significant increases in overdose risk during the subsequent 15 days compared to staying at their previous dose. These findings align with recommendations in the CDC guidelines to prescribe patients the lowest effective opioid dose and avoid major dose increases over short periods of time.^38^

We also found that nearly all patients prescribed daily doses ≥60 MME faced significantly greater overdose risk than patients prescribed stable, low opioid doses regardless of whether their dose increased, decreased, or was stable over 30 days (Table 4a). Patients prescribed ≥60 MME per day who experienced large dose reductions (≥2 categories) were at increased overdose risk compared to patients prescribed stable, high doses; this increased risk was significantly greater for patients prescribed ≥90 MME per day whose opioids were abruptly stopped. These findings suggest that much of the increased overdose risk for these patients is due to their high (≥60 MME per day) baseline opioid dose rather than their 30-day dose trajectory. However, large dose decreases in these patients are associated with increased overdose risk.

Most importantly, our results underscore that, for patients taking ≥90 MME per day, abruptly stopping prescription opioids drastically increases risk of fatal overdose during the subsequent 15 days. These results are consistent with prior studies showing that among patients with likely physical dependence from long-term use, opioid discontinuation is associated with greater overdose risk than dose decreases without discontinuation.^10, 11^ Almost all patients prescribed ≥90 MME per day have physical opioid dependence and so may have sought illicit opioids when their prescriptions were stopped. In contrast, some patients may have had their prescriptions cut off because they had high overdose risk for other reasons (e.g., due to uncontrolled opioid use disorder). Regardless, our results underscore that clinicians should closely monitor all patients prescribed ≥90 MME per day, avoid unilaterally stopping opioids for these patients, obtain patient agreement before considering any dose changes, prescribe naloxone, and screen them for opioid use disorder. These recommendations align with the 2019 US Health and Human Services guidelines on opioid dose reduction and the revised 2022 CDC guidelines, which both urge caution when reducing opioid doses.^38, 39^

Many patients with physical opioid dependence must exert substantial mental and physical effort to successfully reduce their opioid consumption;^40^ these patients appear to remain at high risk for overdose, mental health crisis, and even all-cause mortality for months to years after an opioid dose reduction.^13, 41^ Some protocols for safely reducing patient’s prescribed opioid dose have shown promise.^42, 43^ Transitioning patients to buprenorphine is another potential strategy to reduce overdose risk that will likely become more common since the X-waiver requirement was repealed in December 2022.

We believe our study is the first and largest to estimate the impact of 30-day prescribed opioid dose trajectories on overdose risk across the full range of clinically relevant dose trajectories. Our results are consistent with prior studies examining longer-term dose trajectories and showing that dose increases,^44^ decreases,^9–11^ and dose variability^28^ are all opioid overdose risk factors for patients with physical opioid dependence. Most of these prior studies lacked sufficient sample to examine overdose risks associated with short-term dose trajectories in granular detail.

In addition to findings around prescribed dose, our study examined fatal opioid overdose risk associated with other aspects of controlled substance prescribing. Despite widely publicized concerns about risks associated with carisoprodol, z-drugs, and psychostimulants,^45^ receiving these drug classes was not associated with fatal overdose risk after controlling for other independent variables. Similarly, neither receiving a long-acting opioid formulation nor receiving opioids from multiple prescribers or pharmacies was significantly associated with overdose risk in our model. Prior studies that identified these risk factors did not account for the full range of independent variables in our multivariable model.^46–48^ In contrast, our finding that benzodiazepine co-prescription substantially increases opioid overdose risk is consistent with prior studies on this topic;^49, 50^ additional research on specific patterns and trajectories of co-prescribing associated with overdose risk are needed to inform guidelines for this high-risk patient population.

Our study has limitations. We analyzed older data. Prescribing patterns are much different and rates of high-dose opioid prescribing are much lower today than they were in 2013, so our findings should be interpreted in an appropriate historical context. The inherent risks associated with prescription opioid use are likely stable over time, but replication of our analysis with more recent data can inform specific clinical or policy recommendations. We were unable to examine prescribed opioid dose trajectories for more than one 30-day increment because our data were limited to one calendar year; analyzing data with longer retrospective “look-back” and prospective follow up periods would allow evaluation of how short and long-term dose trajectories jointly impact overdose risk over longer periods of time. Finally, we did not have access to data about patients’ clinical characteristics, about methadone dispensed for addiction treatment, or, most importantly, about why patients’ prescribed dose was changed or abruptly stopped before an overdose. However, these weaknesses are counterbalanced by our use of population-based data from a large US state with a large number of overdose events. Datasets that include clinical and diagnostic data are nearly always restricted to specific insurers or health systems, and so lack either the statistical power or detailed cause-of-death information necessary to evaluate the impact of 30-day changes in prescribed opioid dose on overdose risk.

### Supplementary information

ESM 1 (DOCX 19 kb)

## Data Availability

Data supporting study findings are not available from the authors because data use agreements signed with the California Departments of Justice and Public Health preclude sharing data with third parties.
