# Supplementary material for: Impact of 30-day prescribed opioid dose trajectory on fatal overdose risk: A population-based, statewide cohort study
Source: J Gen Intern Med. 2023 Oct 4;39(3):393–402. doi: 10.1007/s11606-023-08419-6 (PMC10897080; doi:10.1007/s11606-023-08419-6)
Supplement: Supplementary file 1 — (DOCX 19 kb) [file 11606_2023_8419_MOESM1_ESM.docx]

Henry SG et al. “Impact of 30-day prescribed opioid dose trajectory on fatal overdose risk: A population-based, statewide cohort study,” *Journal of General Internal Medicine*, 2023

**Table** **S1.** Estimated associations linking monthly opioid dose with opioid fatal overdose not involving heroin (n = 715) among patients in California, 2013 (N=68,829,519 analyzing samples)

| **Characteristics** | **Prescription opioid**  **fatal overdose (N=605)** | | |
| --- | --- | --- | --- |
|  | **aHR*** | **95% CI** | ***P* value** |
| **Time-fixed covariates** |  |  |  |
| Patient age |  |  |  |
| 12-24 | Ref | -- | -- |
| 25-64 | 1.44 | 0.97-2.14 | 0.073 |
| 65 or more | **0.48** | **0.31-0.74** | **0.001** |
| Patient sex^†^ |  |  |  |
| Female | Ref | -- | -- |
| Male | **1.66** | **1.43-1.92** | **<.001** |
| **Time-varying covariates** |  |  |  |
| **Opioid Dose** |  |  |  |
| Current MME * Previous 30 days MME |  |  |  |
| 0-29*0-29 | Ref | -- | -- |
| 0-29*30-59 | 1.54 | 0.93-2.56 | 0.092 |
| 0-29*60-89 | **4.13** | **2.45-6.96** | **<.001** |
| 0-29*≥90 | **10.19** | **7.06-14.72** | **<.001** |
| 30-59*0-29 | 1.19 | 0.77-1.85 | 0.433 |
| 30-59*30-59 | **1.68** | **1.13-2.51** | **0.011** |
| 30-59*60-89 | 1.80 | 0.76-4.22 | 0.179 |
| 30-59*≥90 | **2.61** | **1.12-6.12** | **0.027** |
| 60-89*0-29 | **1.98** | **1.17-3.35** | **0.011** |
| 60-89*30-59 | 2.01 | 0.95-4.24 | 0.068 |
| 60-89*60-89 | **2.41** | **1.55-3.76** | **0.001** |
| 60-89*≥90 | **3.26** | **1.72-6.17** | **<.001** |
| ≥90*0-29 | **3.81** | **2.50-5.81** | **<.001** |
| ≥90*30-59 | **4.88** | **2.69-8.86** | **<.001** |
| ≥90*60-89 | **2.65** | **1.38-5.08** | **0.003** |
| ≥90*≥90 | **2.61** | **1.77-3.84** | **<.001** |
| **Opioid Characteristics** |  |  |  |
| Current opioid use (Ref=no) | **1.78** | **1.30-2.45** | **<.001** |
| More than one opioid prescription (Ref=0-1) | 1.19 | 0.84-1.68 | 0.332 |
| Any long acting opioid (Ref=no) | 1.21 | 0.86-1.70 | 0.271 |
| Liquid formulation (Ref=no) | 0.85 | 0.40-1.79 | 0.665 |
| Patch or other form formulation (Ref=no) | 1.01 | 0.67-1.51 | 0.966 |
| Opioid type |  |  |  |
| Only hydrocodone or no use | Ref | -- | -- |
| Only oxycodone | 1.35 | 0.98-1.86 | 0.065 |
| Only codeine | 0.54 | 0.22-1.31 | 0.172 |
| Only morphine | **2.21** | **1.37-3.57** | **0.001** |
| Only Buprenorphine | 1.27 | 0.54-2.97 | 0.586 |
| Only Methadone | **3.46** | **2.12-5.64** | **<.001** |
| Only Fentanyl | 1.69 | 0.79-3.63 | 0.180 |
| Only Hydromorphone | **2.71** | **1.49-4.94** | **0.001** |
| Other opioid type | 1.00 | 0.32-3.15 | 0.999 |
| More than one opioid type | 1.34 | 0.91-1.97 | 0.144 |
| **Pharmacy/Prescriber Characteristics** |  |  |  |
| Number of pharmacies dispensing opioid prescriptions (Ref=0-1) | 0.74 | 0.50-1.08 | 0.116 |
| Number of prescribers prescribing opioid prescriptions (Ref=0-1) | 1.30 | 0.90-1.87 | 0.163 |
| **Other Controlled Substances** | |  |  |
| Current * Previous active benzodiazepine prescription | | |  |
| No*No | Ref | -- | -- |
| No*Yes | **3.33** | **2.49-4.45** | **<.001** |
| Yes*No | **2.04** | **1.48-2.83** | **<.001** |
| Yes*Yes | **5.07** | **4.15-6.18** | **<.001** |
| Current Z drugs (Ref=no) | 1.08 | 0.83-1.40 | 0.570 |
| Current carisoprodol (Ref=no) | 0.94 | 0.70-1.25 | 0.648 |
| Current psychostimulants (Ref=no) | 0.71 | 0.44-1.16 | 0.169 |

MME = milligram morphine equivalents; aHR = adjusted hazards ratio; 95% CI = 95% confidence interval

* Time-varying Cox proportional hazards model; model adjusts for all listed covariates; parameters that are statistically significant at the *P* = 0.05 level are in bold.

† Category of unknown sex (n = 17,488, 0.03%) not shown
